# Supplementary material for: Quality of life in older immigrant adults on hemodialysis
Source: PLoS One. 2025 Sep 5;20(9):e0322426. doi: 10.1371/journal.pone.0322426 (PMC12412930; doi:10.1371/journal.pone.0322426)
Supplement: Appendix A — (DOCX) [file pone.0322426.s001.docx]

**Appendix A**

**Interview Guide**

**Opening Paragraph**

This interview is being conducted as part of my dissertation project to graduate with a Doctor of Health Science (DHSc) from the University of Indianapolis. The information you provide will be confidential and used only for this dissertation. No personal identifying information will be shared publicly or published. The questions included in this interview address how you perceive and experience CKD and hemodialysis. You may skip any question you do not feel comfortable asking. My goal is not to have any interruptions during our session. To that end, I am also requesting permission to record this interview. Once the interview is completed, a copy of the transcripts will be sent to you if you would like a copy. This interview will take approximately 45 minutes. Please do not hesitate to ask if you have questions for me at any point in this interview. I want to be sure the questions are straightforward. Do you have any questions for me now before we begin? If you are ready, we can start the interview.

**Demographic**

Participant #: Age: Sex:

Ethnicity: Race: Education of level:

Country of origin: Native language: ………

**Interview Guide**

1. Can you describe where you currently live?
   1. Do you live in a home, community setting, or elsewhere?
2. Who can you rely on for support when you need something?
   1. Who would you call if you needed someone to run an errand for you?
   2. Who would you call if there was an emergency?
3. How long have you lived in the U.S.?
   1. How do you define your immigration status?
   2. How was it like navigating the immigration process and citizenship?
   3. Do you believe your immigration status affects your QoL? How?
4. When were you diagnosed with CKD, and how did you feel about it?
   1. How did you end up being diagnosed with CKD? I mean, what triggered you to seek medical care? Was it a symptom you felt or detected during your routine medical visit?
   2. How did it affect your mental well-being when you received the news that your kidneys could no longer function as usual?

i.) Did you fully understand what CKD entails at the time?

- 1. What has changed in your life since you were diagnosed with CKD?
  2. At what point did you realize that your life has changed?
  3. How did it affect your relationship with your family and loved ones?

1. Did you have access to healthcare before you were diagnosed with CKD?
   1. If yes, how often do you visit your provider? If not, why?
   2. What other health issues do you have before your diagnosis of CKD?
2. How do you define your quality of life with CKD and on hemodialysis?
3. In what ways have CKD and hemodialysis affected your quality of life?
4. Do you feel you need more help now than before to get things done?
5. How long does it take to get hemodialysis access after being diagnosed with CKD?
6. What are some of the things that you do that are most important to you and make you happy?
   1. Are there activities you enjoy but have not engaged in recently, and why?
   2. Do you believe aging restricts you from engaging in activities you enjoy, and why?
   3. How do CKD and hemodialysis affect your day-to-day activities?
7. How do you define your religious, spiritual, and cultural identity?
   1. How has hemodialysis and CKD impacted your religious, spiritual, and cultural practices or identity? i.) Do you feel CKD and hemodialysis restrict you from practicing your religion or culture?
   2. How vital are your spirituality, religiosity, or cultural practices to your happiness or mental health?
   3. Are there any traditional or cultural foods you enjoy eating but could not have due to CKD or hemodialysis?
8. How was your health condition when you first arrived in the U.S.?
   1. What were the events leading to the deterioration of your health condition?
   2. What was the change like in terms of adapting to the food and lifestyle here in the U.S.? i) Were you able to find the same foods you eat while in your native country, or did you change your diet?
9. Some people are bothered by the effects of CKD and hemodialysis on their daily life, while others are not. How much does kidney disease bother you regarding your sexual life, fluid restrictions, ability to work around the house, travels, physical appearance, or depending on healthcare professionals and your family?
10. What is your overall experience with CKD and hemodialysis treatment procedures?
    1. How do you get to your hemodialysis appointments, and how many times a week do you have to go? i.) Do you depend on other people to get to your appointment?
    2. Are the treatment procedures explained to you regarding the type of vascular access you receive your treatment with?
    3. How challenging do you find hemodialysis in terms of sticking to the treatment regimen and being on time for treatment?
    4. What other issues do you encounter with hemodialysis regarding symptoms, for example, pain, constipation, etc.?
11. How do you define your relationship with your hemodialysis care team?
    1. How much support do you receive from your family?
    2. Do you feel you are getting the needed support from your care team and family?
    3. How do you define the communication between you and your healthcare team in terms of fully understanding your treatment schedule and overall procedures?
    4. How involved are you in your treatment decisions? Who makes decisions in terms of your treatment procedures and options?

**Closing Paragraph**

Thank you for taking the time to meet with me today. I appreciate your time and openness. As a reminder, I will contact you after this interview is assessed to get your opinions on my interpretations of what you shared. If you have any questions, please feel free to contact me. Again, my name is X, and my phone number is X.
