# Supplementary material for: Quality of life in older immigrant adults on hemodialysis
Source: PLoS One. 2025 Sep 5;20(9):e0322426. doi: 10.1371/journal.pone.0322426 (PMC12412930; doi:10.1371/journal.pone.0322426)
Supplement: Appendix B — (DOCX) [file pone.0322426.s002.docx]

**Appendix B**

**Cognitive Assessment** **Tool**

**Short Blessed Test (SBT)1**

Patient: ________________ DATE: ____________ Age: ___________

“Now, I would like to ask you some questions to check your memory and concentration. Some of them may be easy and some of them may be hard.”

1. What year is it now? ______________ Correct Incorrect (0) (1)

2. What month is it now?_____________ Correct Incorrect (0) (1)

Please repeat this name and address after me:

John Brown, 42 Market Street, Chicago

John Brown, 42 Market Street, Chicago

John Brown, 42 Market Street, Chicago

(underline words repeated correctly in each trial)

Trials to learning________(can’t do in 3 trials = C)

Good, now remember that name and address for a few minutes.

3. Without looking at your watch or clock, tell me about what time it is.

(If the response is vague, prompt for a specific response) Correct Incorrectly

(within 1 hour) _______ (0) (1)

Actual time: _____________

4. Count aloud backwards from 20 to 1 0 1 2 Errors (Mark correctly sequenced numerals)

If the subject starts counting forward or forgets the task, repeat instructions and score one error

20 19 18 17 16 15 14 13 12 11

10 9 8 7 6 5 4 3 2 1

5. Say the months of the year in reverse order. If the tester needs to prompt with the last name of the month of the year, one error should be scored (Mark correctly sequenced months)

D N O S A JL JN MY AP MR F J 0 1 2 Errors

6. Repeat the name and address I asked you to remember.

(The thoroughfare term (Street) is not required)

(John Brown, 42 Market Street, Chicago)

0 1 2 3 4 5 Errors

_____, ______, ___, ___________, ________

Check the correct items.

USE ATTACHED SCORING GRID & NORMS

A spontaneous self-correction is allowed for all responses without counting as an error.

1. What is the year?

Acceptable Response: The exact year must be given. An incomplete but correct numerical response (e.g., 01 for 2001) is acceptable.

2. What is the month?

Acceptable Response: The exact month must be given. A correct numerical answer is acceptable (e.g., 12 for December).

3. The clinician should state: “I will give you a name and address to remember for a few minutes. Listen to me say the entire name and address and then repeat it after me.”

It is important for the clinician to carefully read the phrase and emphasize each item of the phrase. There should be a one second delay between individual items. The trial phrase should be re-administered until the subject is able to repeat the entire phrase without assistance or until a maximum of three attempts. If the subject is unable to learn the phrase after three attempts, a “C” should be recorded. This indicates the subject could not learn the phrase in three tries.

Whether or not the trial phrase is learned, the clinician should instruct “Good, now remember that name and address for a few minutes.”

4. Without looking at your watch or clock, tell me about what time it is?

This is scored as correct if the time given is within plus or minus one hour. If the subject’s response is vague (e.g., “almost 1 o’clock), they should be prompted to give a more specific response.

5. Counting. The instructions should be read as written. If the subject skips a number after 20, an error should be recorded. If the subject starts counting forward during the task or forgets the task, the instructions should be repeated and one error should be recorded. The maximum number of errors is two.

6. Months. The instructions should be read as written. To get the subject started, the examiner may state “Start with the last month of the year. The last month of the year is________________.” If the subject cannot recall the last month of the year, the examiner may prompt this test with “December”; however, one error should be recorded. If the subject skips a month, an error should be recorded. If the subject starts saying the months forward upon initiation of the task, the instructions should be repeated, and no error should be recorded. If the subject starts saying the months forward during the task or forgets the task, the instructions should be repeated, and one error should be recorded. The maximum number of errors is two.

7. Repeat. The subject should state each item verbatim. The address number must be exact (i.e., “4200” would be considered an error for “42”). For the name of the street (i.e., Market Street), the thoroughfare term is not required to be given (i.e., Leaving off “drive” or “street”) or to be correct (i.e., Substituting “boulevard” or lane”) for the item to be scored correctly.

8. The final score is a weighted sum of individual error scores. Use the table on the next page to calculate each weighted score and sum for the total.

**Final SBT Score & Interpretation**

| Item # | Errors (0 - 5) | Weighting Factor | Final Item Score |
| --- | --- | --- | --- |
| 1 X 4 |  |  | 1 X 4 |
| 2 X 3 |  |  | 2 X 3 |
| 3 X 3 |  |  | 3 X 3 |
| 4 X 2 |  |  | 4 X 2 |
| 5 X 2 |  |  | 5 X 2 |
| 6 X 2 |  |  | 6 X 2 |
|  |  |  | Sum Total _________  (Range 0 – 28) |

**Interpretation**

A screening test in itself is insufficient to diagnose a dementing disorder. The SBT is, however, quite sensitive to early cognitive changes associated with Alzheimer’s disease. Scores in the impaired range (see below) indicate a need for further assessment. Scores in the “normal” range suggest that a dementing disorder is unlikely, but a very early disease process cannot be ruled out. More advanced assessment may be warranted in cases where other objective evidence of impairment exists. In the original validation sample for the SBT (Katzman et al., 1983), 90% of normal scores 6 points or less. Scores of 7 or higher would indicate a need for further evaluation to rule out a dementing disorder, such as Alzheimer’s disease. Based on clinical research findings from the Memory and Aging Project, the following cut points may also be considered:

- 0 – 4 Normal Cognition
- 5 – 9 Questionable Impairment (evaluate for early dementing disorder)
- 10 or more Impairment Consistent with Dementia (evaluate for dementing disorder).
