# Supplementary material for: Quality of life in older immigrant adults on hemodialysis
Source: PLoS One. 2025 Sep 5;20(9):e0322426. doi: 10.1371/journal.pone.0322426 (PMC12412930; doi:10.1371/journal.pone.0322426)
Supplement: Appendix C — (DOCX) [file pone.0322426.s003.docx]

Are you an older immigrant with chronic kidney disease? We would like to learn about your experience of living with chronic kidney disease in the U.S. and its impact on your quality of life.

Be A Voice for Immigrants with Chronic Kidney Disease


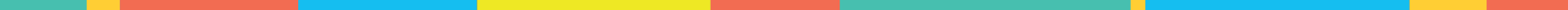


**Volunteers Needed
for a Research Study on Chronic Kidney Disease**

You May Qualify If You

- Were born and raised outside of the U.S. and migrated to the U.S. as an adult.
- Aged 65 or older living in the U.S.
- Diagnosed with Stage 5 chronic kidney disease
- On hemodialysis for at least 3 months
- Able to read, write, and understand English
- **Participation Involves**
- Taking part in a one-time 45-minute interview.
- After the interview, reviewing the transcript of the interview to check for accuracy.
- Allowing the interview to be recorded (audio and/or video)

**We Want to Hear From You!**

- Participation is voluntary.
- All selected study participants will receive a $25 Amazon gift card.

**FOR MORE INFORMATION**

**Please contact Demba Keita at 518-709-9896, email** [**keitad@uindy.edu**](mailto:keitad@uindy.edu)
