# Supplementary material for: Quality of life in older immigrant adults on hemodialysis
Source: PLoS One. 2025 Sep 5;20(9):e0322426. doi: 10.1371/journal.pone.0322426 (PMC12412930; doi:10.1371/journal.pone.0322426)
Supplement: Appendix D — (DOCX) [file pone.0322426.s004.docx]

**CONSENT TO PARTICIPATE IN A RESEARCH STUDY**

**STUDY TITLE:** Quality of Life in Older Immigrant Adults on Hemodialysis

**PRINCIPAL INVESTIGATOR:** Elizabeth S. Moore, PhD

**STUDENT INVESTIGATOR:** Demba Keita

**CONTACT DETAILS:** Call: (317) 788-6107

Email: moorees@uindy.edu

**ELIGIBILITY:** You are eligible to participate in the study if you meet the following criteria:

- In immigrant to the United States
- 65 years or older
- Stage 5 chronic kidney disease (CKD
- Live full-time in the United States
- Receive hemodialysis for at least the last 3 months.
- Able to read, write, and understand English.
- Do not have a cognitive impairment.

Participation in the study is voluntary. Please read the following information below carefully so you can make an informed decision about whether you would like to volunteer.

**PURPOSE:** This study explores how older immigrant adults perceive the influence of living with stage 5 CKD on their quality of life (QoL). Also, the study will explore how older immigrant adults describe the impact of living with stage 5 CKD and hemodialysis on their cultural beliefs and practices.

**PROCEDURES:** If you volunteer to participate in the study, the student researcher (D. K.) will ask you to do the following:

- Over the phone or through Zoom or Google Meet, answer 6 questions from a Short Blessed Test questionnaire. The questions will ask you about your memory and concentration.
- Answer questions about how living with stage 5 CKD influences your QoL and how living with stage 5 CKD impacts your cultural beliefs and practices. The interview will take place over the phone or with Zoom or Google Meet
- Allow the researcher to audiotape and/or video tape the interview.
- Allow the research to contact you by the phone or with Zoom or Google Meet in two weeks after the interview to share a summary of the information collected from you to ensure it is an accurate interpretation.

**DURATION:** Participation will take approximately 45 minutes to complete.

**RISKS AND DISCOMFORT:** Participating in this study involves minimal risks. You may feel uncomfortable discussing specific topics, but I will do my best to create a safe and supportive environment.

**BENEFITS:** You will not directly benefit from your participation in the research.

**COMPENSATION:** You will not receive compensation for participation, but you will receive a $25 Amazon gift card to thank you for taking part in the study.

**CONFIDENTIALITY:** The results of this study may be published in a scholarly book or journal, presented at professional conferences, or used for teaching purposes. However, only aggregate data will be used. Personal identifiers will not be used in publication, presentation, or teaching materials. Instead, pseudonyms or codes will be used.

**DATA USAGE IN FUTURE STUDIES:** It is possible that de-identified data from this study could be used for future research or shared with other researchers for use in studies without additional informed consent. De-identified means that any codes and personal information that could identify you will be removed before the data is shared.

**PARTICIPANT RIGHTS:** You can choose whether you want to be in this study, and you may withdraw your consent and discontinue participation at any time. Whatever decision you make, there will be no penalty to you and no loss of benefits or incentives to which you were otherwise entitled. Participation will not affect your relationship with the University of Indianapolis, the primary researcher, or the dialysis center. You may refuse to answer any question/s you do not want to answer and remain in the study.

**REQUEST FOR MORE INFORMATION:** If you have any questions, comments, or concerns about the research, you can talk to one of the researchers. Please contact the Principal Investigator, Elizabeth S. Moore, by phone at 1 (317) 788-6107 or by email at [moorees@uindy.edu](mailto:moorees@uindy.edu).

If you have any questions about your rights as a research participant, or you have concerns or suggestions and want to talk to someone other than the researchers, you may contact the Director of the Human Research Protection Program by either emailing [hrpp@uindy.edu](mailto:hrpp@uindy.edu) or calling 1 (317) 781-5774.

**INFORMED CONSENT:** If you consent to participate in this study, then you affirm that you satisfy inclusion criteria, that you have read and understood this document, and that your consent is voluntary. Before the beginning of the interview, the researcher will ask you to confirm verbally that you consent to participate and that you consent to the interview being audio and/or videotaped.
