# Supplementary material for: Quality of life in older immigrant adults on hemodialysis
Source: PLoS One. 2025 Sep 5;20(9):e0322426. doi: 10.1371/journal.pone.0322426 (PMC12412930; doi:10.1371/journal.pone.0322426)
Supplement: Attachment — Submitted filename: Rebuttal Letter.docx [file pone.0322426.s006.docx]

Dear Editor-in-Chief,

Thank you for your constructive feedback on our manuscript titled *“Quality of Life in Older Immigrant Adults on Hemodialysis”*. We deeply appreciate the time and effort that you and the reviewers have invested in evaluating our work. Below, we provide a detailed response to each of the points raised and outline the revisions we have made to address them.

**Reviewer #1:**

1. **Sample Size Justification**:
   - *Comment*: The reviewer pointed out that we did not provide a clear rationale for our sample size of 10-12 participants.
   - *Response*: We have revised the manuscript to provide a detailed explanation of our sample size. The sample was determined based on the information power of the study, which ensures that a sufficient number of participants were included to capture the depth and variability of the experiences of older immigrant adults undergoing hemodialysis. We have added this explanation in the Methods section on page 19.
2. **Data Availability**:
   - *Comment*: The reviewer noted that the data availability statement lacks clarity on where the data can be accessed and recommended a more comprehensive data-sharing plan.
   - *Response*: We have updated the Data Availability Statement to include specific information about where the data can be accessed once ethical and privacy concerns are addressed. The data will be deposited in a public repository (e.g., [institutional repository name or link]) and can be accessed upon request. We have included this information on page 28 of the revised manuscript.
3. **Discussion of Cultural Practices and Health**:
   - *Comment*: The reviewer suggested that we provide a clearer exploration of the intersection between QoL and cultural practices, especially with regards to specific rituals like Ramadan fasting.
   - *Response*: We have expanded the discussion on cultural practices and their impact on QoL in the revised manuscript (page 23). We provide a deeper analysis of how religious practices, such as fasting during Ramadan, present challenges for patients on hemodialysis and how healthcare providers can offer culturally sensitive support.
4. **Health and Illness Perceptions**:
   - *Comment*: The reviewer recommended further elaboration on how participants' perceptions of CKD and dialysis treatment align with their cultural beliefs.
   - *Response*: In the revised manuscript (page 17), we have added a section discussing how participants’ cultural beliefs shape their understanding of CKD and its treatment. We specifically highlight how cultural values influence patients' attitudes towards dialysis and their adherence to treatment regimens.
5. **Use of Direct Participant Quotes**:
   - *Comment*: The reviewer mentioned that some participant quotes were too generalized and suggested using more detailed and personalized quotes.
   - *Response*: We have replaced the more generalized quotes with more personalized, detailed quotes that reflect the unique experiences of each participant. For example, we have now included quotes that describe emotional responses in greater depth, which better illustrate the variation in experiences among participants. These changes are reflected throughout the revised manuscript.
6. **Clinical Implications of Findings**:
   - *Comment*: The reviewer suggested expanding the clinical implications of the findings and offering specific, actionable recommendations for healthcare providers.
   - *Response*: We have added a section in the Discussion where we provide concrete, actionable recommendations for healthcare providers to improve the QoL of older immigrant adults on hemodialysis. These include culturally competent care practices, spiritual support, and personalized treatment plans.

**Reviewer #2:**

1. **Formatting and Structure**:
   - *Comment*: The reviewer recommended structural revisions to meet the journal’s submission guidelines, including adjustments to the number of headings, continuous line numbering, and citation formatting.
   - *Response*: We have revised the manuscript to comply with the journal’s guidelines. The manuscript now follows the three-level heading structure, continuous line numbering has been applied throughout, and the references are numbered in the order of their appearance. We have also formatted the references according to the Vancouver style.
2. **Abstract**:
   - *Comment*: The reviewer suggested improving the clarity and structure of the abstract by including a concise description of the methodology and key findings.
   - *Response*: The abstract has been revised to include more detail about the methodology and key findings. We have clarified the research design, participant selection process, and the major themes that emerged from the study, ensuring that the abstract provides a comprehensive summary of the manuscript’s content.
3. **Keywords**:
   - *Comment*: The reviewer noted that three of the five provided keywords were not indexed in the MeSH database.
   - *Response*: We have updated the keywords to include those indexed in the MeSH database. The new keywords now better align with the terms used in the literature and will help improve the visibility of the manuscript.
4. **Chapters 1 and 2 (Introduction and Literature Review)**:
   - *Comment*: The reviewer requested a revision of these sections to reduce repetition and improve clarity.
   - *Response*: We have condensed the Introduction and Literature Review sections to remove redundant information. The revised sections now focus on providing a concise overview of the relevant background and research gaps, enhancing the overall flow and clarity of the manuscript.
5. **Chapter 3 (Methods)**:
   - *Comment*: The reviewer suggested that some content in the Methods section be relocated for better clarity, particularly regarding definitions and sample descriptions.
   - *Response*: We have reorganized the Methods section by relocating the theoretical definitions to the theoretical framework and streamlining the discussion of the sample under the “Sampling and Recruitment” section. Additionally, we have ensured that no content is repeated in the ethical statement and sampling descriptions.
6. **Tables**:
   - *Comment*: The reviewer noted that the tables were overly simplistic and suggested enhancing them to improve the depth and quality of analysis.
   - *Response*: We have revised the tables to include more detailed information. For example, we have added specific data points and enhanced the layout to present the findings more effectively. The revised tables provide a clearer representation of the study's findings and support the manuscript’s analysis.

**Additional Requirements:**

1. **Response to Reviewer/Editor Comment**

**Comment**: "Please ensure that your manuscript meets PLOS ONE's style requirements, including those for file naming. The PLOS ONE style templates can be found at [link to main body template] and [link to title, authors, and affiliations template]."

**Response**:
I have reviewed the PLOS ONE style requirements and have thoroughly revised our manuscript to align with the guidelines provided. I used the PLOS ONE style templates for both the main body of the manuscript and the title/authors/affiliations section, as outlined in the links provided. All required sections have been formatted accordingly, including the abstract, introduction, methods, results, discussion, and references, ensuring consistent use of fonts, headings, and text size.

Additionally, we have ensured that the file naming conventions specified by PLOS ONE are strictly followed, with all sections clearly labeled (e.g., "Manuscript.docx," "Figure1.tif," etc.). We believe these revisions fulfill the style requirements, and the manuscript is now in full compliance with PLOS ONE’s submission guidelines.

2. **Ethics Statement**:
   - *Response*: We have included the full ethics statement in the Methods section, including the name of the IRB that approved the study, details about the verbal consent process, and the documentation and witness procedures for consent.
3. **Data Availability Statement**:
   a. *Response*: The Data Availability Statement has been revised to confirm that the minimal data set required to replicate the study findings will be made available upon request.

**Conclusion:**

We have carefully addressed all of the comments and suggestions provided by the reviewers. The manuscript has been revised to improve clarity, structure, and alignment with PLOS ONE’s submission guidelines. We believe the revisions have enhanced the manuscript and look forward to your feedback.

Thank you for your time and consideration.

Sincerely,
Demba Keita, MD, DHSc, MPH
Epidemiological Supervisor
Sullivan County Department of Public Health
[Demba.Keita@sullivanny.gov](mailto:Demba.Keita@sullivanny.gov) or [demba.aja84@gmail.com](mailto:demba.aja84@gmail.com)
